# Dental education in primary care: 14 years of Peninsula Dental School

**DOI:** 10.1038/s41415-022-4554-6

**Published:** 2022-08-12

**Authors:** Christopher Tredwin, Sally Hanks, Rob Witton, Ewen McColl, Cathy Coelho

**Affiliations:** 41415141187001 Professor of Restorative Dentistry and Head of School, Peninsula Dental School, University of Plymouth, John Bull Building, 16 Research Way, Plymouth Science Park, Plymouth, Devon, PL6 8BU, UK; 41415141187002 Professor of Primary Care Dentistry, Associate Head of School Teaching and Learning, Peninsula Dental School, University of Plymouth, John Bull Building, 16 Research Way, Plymouth Science Park, Plymouth, Devon, PL6 8BU, UK; 41415141187003 Professor of Community Dentistry, Peninsula Dental School, University of Plymouth, Derriford Dental Education Facility, Plymouth Science Park, Research Way, Plymouth, PL6 8BT, UK; 41415141187004 Director of Clinical Dentistry, Peninsula Dental School, University of Plymouth, Derriford Dental Education Facility, Plymouth Science Park, Research Way, Plymouth, PL6 8BT, UK; 41415141187005 Associate Professor in Clinical Dental Education, Lead of the Simulated Dental Learning Environment, Peninsula Dental School, University of Plymouth, Derriford Dental Education Facility, Plymouth Science Park, Research Way, Plymouth, PL6 8BT, UK

## Abstract

Peninsula Dental School, established in 2006, was the UK's first new dental school in 40 years. It had the freedom to develop a completely new dental education curriculum planned on pedagogic thinking, designed to equip the dental care professionals of the twenty-first century. This was based on three distinct pillars: professionalism (developing a student's trust in their own autonomy); dental skills of the highest order (not just technical skills but also communication skills); and social engagement. As such, a truly innovative approach to dental education was created that has strong roots in evidence.

This paper describes the University of Plymouth Peninsula Dental School's achievements against these initial objectives under the following areas: training in primary care; a novel spiralling integrated curriculum and assessments; facilities reaching out to deliver patient care; bringing meaningful patient contact to students from the earliest months of their course; embedding community engagement within the curriculum; development of Peninsula Dental Social Enterprise; and team working, training a variety of dental care profession students side by side.

The University of Plymouth Peninsula Dental School, working with all its partners, has successfully pioneered and delivered significant changes in the field of education and continues to strive to further develop these and more for the future.

## Key points

- An insight into the development of a new UK dental school.
- A focus on dental education based in primary care, reaching out to deliver patient care, embedding community engagement in a curriculum and team working of dentists and dental care professionals side by side.
- An overview of the benefits of a social enterprise clinical placement model.

## Introduction

When the agreement was signed for its creation in 2006, Peninsula Dental School (PDS) was established as the UK's first new dental school for 40 years. This was the result of a successful bid to government which sought to alleviate pressures on dental care provision by producing more dental care professionals, while also seeking new approaches to dental education and care delivery.

The successful bid gave the University the freedom to develop a completely new dental education curriculum planned on pedagogic thinking and based on three distinct pillars: professionalism (developing a student's trust in their own autonomy); dental skills of the highest order (not just technical skills but also communication skills); and social engagement. As such, a truly innovative approach to dental education was created that has strong roots in evidence and which has been designed to equip the dental care professionals of the twenty-first century. Importantly, the new approach enabled the University to deliver its core mission and values in this crucial area of health training.

This paper describes the University of Plymouth PDS's achievements against these initial objectives under the following areas:

- Training in primary care
- A novel spiralling integrated curriculum and assessments
- Facilities reaching out to deliver patient care
- Bringing meaningful patient contact to students from the earliest months of their course
- Embedding community engagement within the curriculum
- Development of Peninsula Dental Social Enterprise (PDSE)
- Team working, training a variety of dental care profession students side by side.

## Training in primary care

**Fig. 1:**
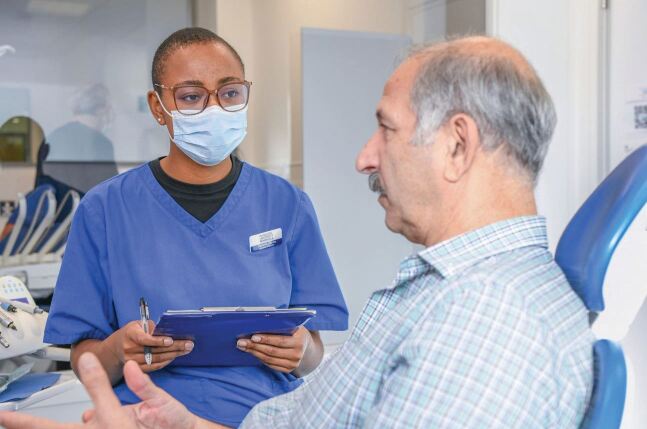
Training taking place in primary care in a dental education facility

**Fig. 2:**
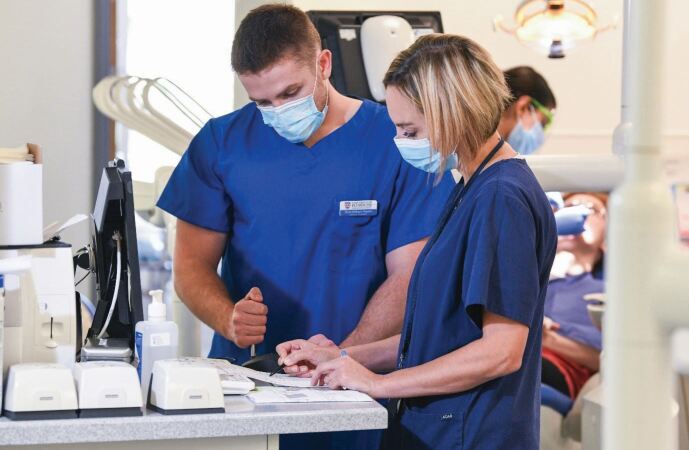
Training taking place in primary care in a dental education facility

A curriculum and training has been designed and implemented based in primary care and on the relational and communication aspects of dentistry, in addition to the technical. This has resulted in dental care professionals who are trained in an environment that mirrors that in which most of them will work - 97% of dentists practise in the primary care setting. The School was the first to break free of the medical model, where training is mainly based in secondary care (an innovation now being adopted by health profession courses across the University) and there are other dental schools seeking guidance and advice on best practice from the institution. More than 500 dental professionals have graduated from the University to date (Figures 1 and 2).

## A novel spiralling integrated curriculum and assessments

PDS offers a five -year undergraduate Bachelor of Dental Surgery (BDS); a three -year integrated Bachelor of Science in Dental Therapy and Hygiene (DTH); a six-year BDS; a four-year DTH with integrated foundation year; a face-to face Masters Degree (Restorative, Periodontics, Endodontics, Oral Surgery and Orthodontics); and a distance learning Masters Degree in Restorative Dental Science.

All teaching and learning activities are patient- and student-centred and are specifically designed for students to experience authentic and contextual learning based around patient care. The opportunities in PDS ensure students are fully prepared for a primary care career, in addition to creating the foundations on which to develop their knowledge and skills in more specialist areas.

Small-group, enquiry-based learning is used as a central opportunity to integrate learning across the curriculum and this is supported by a blend of small- and large-group, interactive and practical sessions. Under the facilitation of a subject expert (general dental practitioner) and following appropriate preparation, participants will be expected to question, critically analyse, evaluate, present and discuss a range of topics. Such learning promotes critical thinking, clinical problem solving and lifelong professional learning.^1^

**Fig. 3:**
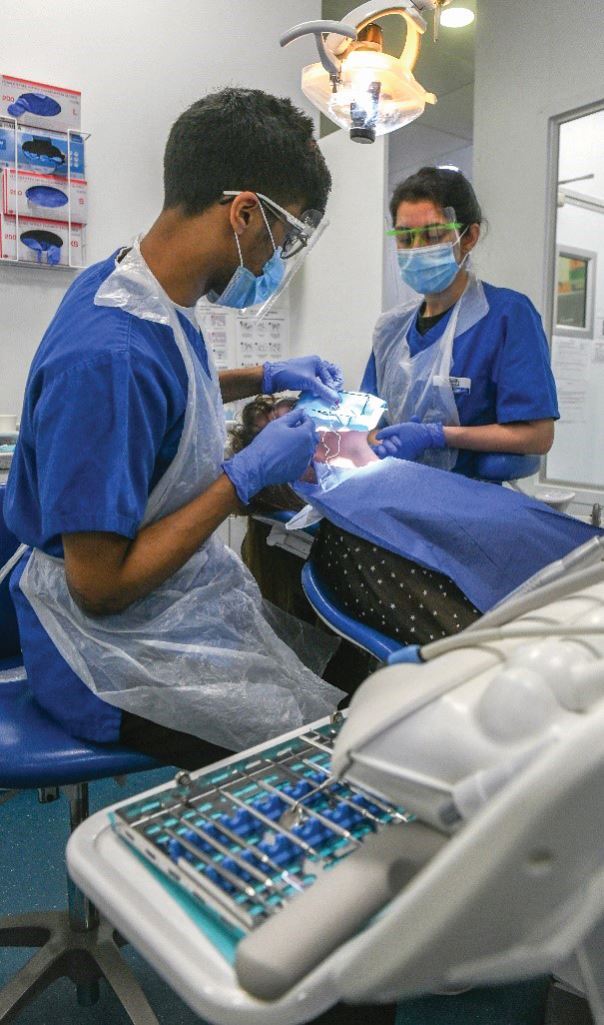
Final year students undertaking a clinical assessment

Clinical assessments follow the route of competence to proficiency to capability as they move from the simulated to clinical environment and towards independent performance. Figure 3 shows final year students undertaking clinical assessments.

In Years 3, 4 and 5 BDS and Years 2 and 3 DTH, students sit the applied dental (therapy) knowledge (ADTK) progress tests that are based on the expected knowledge of a newly qualified dentist or therapist. The progress test questions are set at the standard of knowledge expected from a newly qualified dentist/therapist, that is, at the level of a new graduate. As each student advances through the programme, increasing correct scores and fewer 'I don't know' responses indicate progressive growth in knowledge. The content and the method of assessment underpin authentic learning goals (for example, negative marking embeds the clinical reality that 'don't know' is better than guessing and making the wrong decision clinically; and returning to previously answered questions to reflect and refine if necessary mirrors the clinical importance of continuous re-evaluation to ensure revision of treatment plans where appropriate).^2^^,^^3^

The overall learning, teaching and assessment approaches employed within these programmes aim to improve health outcomes and to facilitate real change in the quality of clinical care and health for individuals and the wider community, regionally, nationally and globally. The underlying strategy embeds the move from competence to capability as students become more clinically independent and prepared for practice as they approach the end of their training.^4^^,^^5^

## Facilities reaching out to deliver patient care

**Fig. 4:**
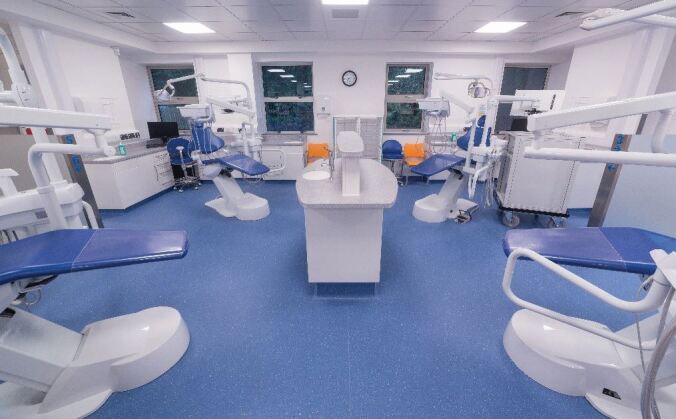
Layout of a bay in a dental education facility

**Fig. 5:**
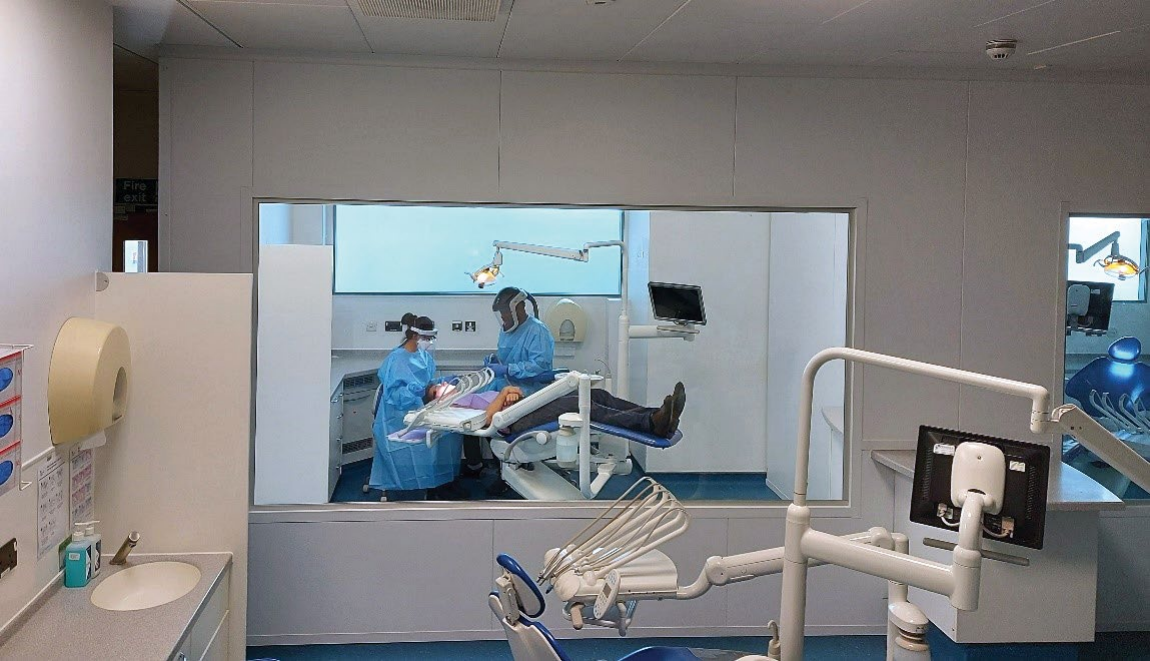
Purpose-built aerosol generating procedure pods

Four purpose-built dental education facilities (DEFs), two in Plymouth and one each in Exeter and Truro, where dental care profession students treat NHS patients under the supervision of qualified dental care professionals, are the product of a total combined investment of around £20 million. This has revolutionised dental health care education delivery, while also providing access to NHS dental treatment for over 50,000 patients since the DEF programme opened in 2008 - many from areas of need and social deprivation in Devon and Cornwall and who may not have had that access before. Examples of the layout of a bay in the dental education facilities is shown in Figure 4 and the adaption of these and the building of special pods during the COVID-19 pandemic is shown in Figure 5.

## Bringing meaningful patient contact to students from the earliest months of their course

Early patient contact for students results in well-rounded dental care professionals who are valued by the profession and whose excellent clinical skills are matched by their 'people' skills. The success of the approach has seen more dental schools across the UK introducing their students to patients early.

Early patient contact commencing in Year 1 remains one of the standout characteristics of our programme and plays an important role in developing students' communication and team working skills and understanding the ethos of patient-centred care. Through the later years, students gain a deeper understanding of the patient journey through both primary, secondary and specialist dental care via placements in secondary care and specialist clinical contexts. In final year, students are four days on clinic with one academic day a week and are provided with a thorough grounding for contemporary practice through detailed preparation toward the skills required as a foundation dentist immediately after graduation. Year 5 BDS and Year 3 DTH sees the transition from supervised student to independent practitioner, supported through work-based learning in the primary care environment and the unique academic resource Daybook, developed by Peninsula staff.^1^

## Embedding community engagement within the curriculum

Embedding community engagement in the curriculum has brought significant benefits, including oral health awareness, care and treatment to a wide variety of community groups (including young parents with infants, adults with learning/physical disabilities, older people in care homes, the homeless, people with substance abuse problems, asylum seekers, etc). It results in dental care professionals who have true empathy for their patients, understand the breadth of community and have honed their communication and patient management skills before treating patients.

Learning from and alongside community groups has given students the unique opportunity to gain an understanding of the specific needs and demands of some groups of people that they might not normally have mixed with.^6^^,^^7^^,^^8^ Their inter-professional engagement modules allow for a significant amount of time being spent with disadvantaged communities and other members of the wider healthcare team. As the modules spiral in complexity through the years, there is continuous development of life skills and growth in professionalism, social accountability and understanding of others.^9^ The exceptional partnership between the school and its NHS arm, Peninsula Dental Social Enterprise, guarantees Peninsula Dental School students are well prepared and equipped for their professional career.

## Development of Peninsula Dental Social Enterprise

The creation of the PDSE in 2013 provided a 'corporate' structure for the management and development of the dental learning clinical environment; the structure for a consistent and sustainable community engagement programme and a vehicle to manage clinical and professional governance.

The PDSE is a unique innovation. Created in response to changes in the NHS landscape, it has brought together DEFs in Plymouth, Truro and Exeter under one umbrella, where students from the University treat NHS patients under supervision, alongside providing dental treatment and outreach services to local communities. PDSE is a social enterprise community interest company (CIC) and it is the only model of its type supporting a university dental school in the UK.^10^ As a CIC, everything it does is guided by its community purpose to which its assets and profits are dedicated.

The clinical model of PDSE has enabled the University to more closely align its clinical curriculum with the oral healthcare needs of local communities through a combination of outreach work and research-informed service design.^11^^,^^12^^,^^13^ The DEFs are located in or close to areas of high deprivation and are intended to enable access to dental treatment for disadvantaged and socially marginalised people who frequently suffer from poor oral health.

Its operation has been designed to maximise the community benefit that arises as a by-product of the University's core business in dental education. In so doing, it works closely with the NHS, Health Education England and community organisations in Devon and Cornwall to support oral health. In a typical year, it provides dental care to over 5,000 people who would otherwise find it difficult or impossible to access dental care.^14^ It has also developed a range of dedicated dental services and programmes for disadvantaged people, including those experiencing homelessness, those with learning disabilities, children in care, asylum seekers and refugees and families living in poverty. These initiatives are transformative for patients and deliver social impact and longer-term benefits to the health system.^11^^,^^12^

The economic and social value it generates has been independently assessed and is additional to that derived through the University's core education business. In 2020/21, dental treatment provided quality-of-life gains at up to £2.9 million and an additional social value of £1.1 million, highlighting the impact that staff and students can generate for society in the course of undertaking their core work.^15^ The social enterprise has established itself as an innovative and award-winning organisation. Blurring the lines between curriculum and community, providing graduates with a broader range of skills and delivering direct benefits to the community, brings 'added value' to the mission of dental schools. It was awarded the Social Enterprise Mark two months after launch and has won numerous regional and national awards in the education, NHS, dental, business and social enterprise sectors.

## Training a variety of dental care profession students side by side

The training of dental, dental nurse and dental therapy and hygiene students side by side results in dental care professionals who have experience of, and who understand the dynamics of, the dental care team: an approach also being adopted by other dental schools.

**Fig. 6:**
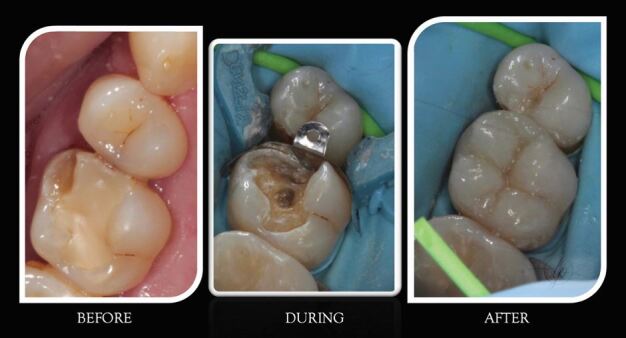
Shared care working and examples of replacement of restorations carried out by a dental therapy student (image courtesy of Lorena Pivoda, now GDC-registered dental therapist)

Interprofessional learning is a key theme throughout, with BDS and DTH students working alongside one another in academic and clinical environments. They are able to complete 'shared care' for patients, thus learning from, with and about one another, to support each other to be successful in their studies, as well as preparing them for the team-working element of dental practice following graduation.^16^ Figure 6 shows an example of work undertaken by the students.

## Conclusions

The establishment of the first new dental school in the UK for over 40 years in 2006 allowed an opportunity to look at dental education and ways to adapt it for the twenty-first century. The University of Plymouth PDS, working with all its partners, has successfully pioneered and delivered significant changes in the field of education and continues to strive to further develop these and more for the future.
